# Effects of the Maillard Reaction on the Structural and Functional Properties of Camel Whey Protein

**DOI:** 10.3390/foods14132201

**Published:** 2025-06-23

**Authors:** Ying Liu, Chunyan Ran, Hongyi Zhang, Yaqi Cheng, Minaer Huanbieke, Yuying Liu, Jie Yang, Yuqing Mei, Yang Qu

**Affiliations:** College of Life Science and Technology, Xinjiang University, Urumqi 830046, China; liuying2020@xju.edu.cn (Y.L.); kally_51@163.com (C.R.); zhy2781954495@163.com (H.Z.); moon_532@163.com (Y.C.); minaerhuan@163.com (M.H.); 18724921090@163.com (Y.L.); yangjie234@xju.edu.cn (J.Y.); myq6661314@163.com (Y.M.)

**Keywords:** camel whey protein, Maillard reaction, functional properties, structural properties

## Abstract

Consumer demand for dairy products like cheese and curds has resulted in a rise in whey production, which has caused significant waste and environmental issues. For this reason, improving the functional characteristics of whey proteins and their usage value are essential. In this study, camel whey protein–galactose conjugates (CWP-Gal) and camel whey protein–glucose conjugates (CWP-Glu) were prepared through the Maillard reaction, and their structural and functional properties were characterized. Improvements in solubility of 14.90% and 8.17%, emulsification activity of 15.53% and 13.64%, and foaming capacity of 113.95% and 106.03% were demonstrated by CWP-Gal and CWP-Glu in comparison to camel whey protein (CWP). Circular dichroism analysis revealed secondary structure alterations in CWP-Gal and CWP-Glu compared to CWP. SDS-PAGE, FT-IR, and intrinsic fluorescence spectroscopy all verified that sugar molecules and proteins were covalently conjugated. SEM analysis revealed that the conjugates had a more sparsely packed microstructure. The results demonstrate that CWP-Gal exhibits enhanced structural stability and superior functional properties, providing a scientific basis for its potential utilization in the food industry.

## 1. Introduction

Whey proteins are by-products of the manufacturing process of cheese and casein dairy products. In the last several years, as the desire of consumers for goods such as cheese and curdled milk products has increased, so has the amount of waste whey produced [1]. Within the EU, annual whey production amounts to approximately 40 million tons, and there is a surplus of 13 million tons of cheese whey [2]. The substantial volumes of waste whey produced present potential environmental concerns, leading to their treatment as pollutants [3]. The primary factors contributing to this challenge are the high cost of whey protein isolation, which is unaffordable for small and medium-sized businesses (SMEs), and the low profitability of its low-value-added use (e.g., animal feed), which restricts its applicability [4]

**Scheme 1:**
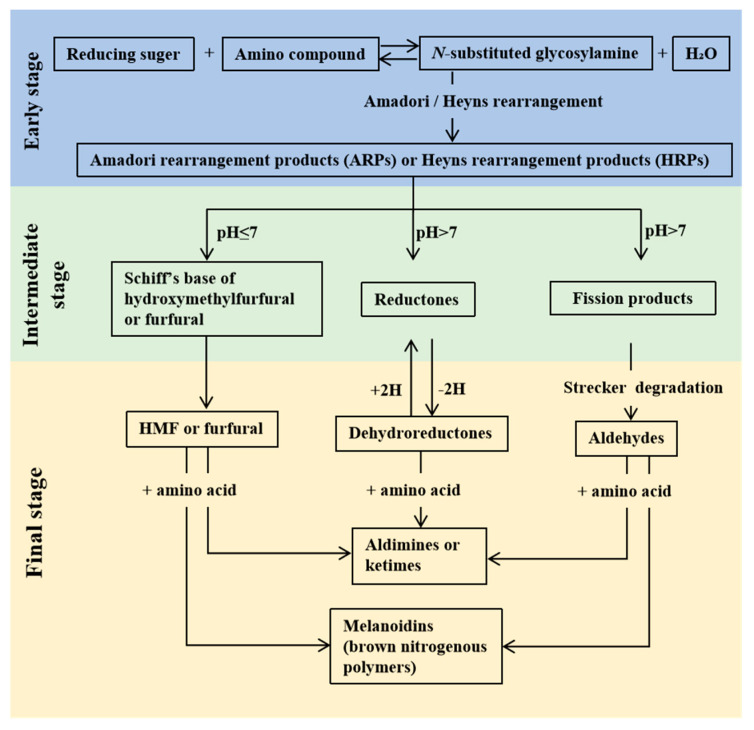
Maillard reaction scheme adapted from Nooshkam [9].

Numerous studies have shown that whey proteins can be modified chemically, enzymatically, and physically to improve their functional qualities [5,6,7]. Among these techniques, the Maillard reaction (MR), which involves covalently linking sugar molecules and whey proteins, has attracted a lot of interest. MR proceeds through three distinct stages: initial, intermediate, and final. In the initial stage, amino groups (e.g., ε-NH_2_ of lysine) in proteins react with carbonyl groups in reducing sugars via condensation to form reversible Schiff bases (-C=N-), which subsequently rearrange to yield stable Amadori compounds (from aldoses via 1,2-enolization) or Heyns compounds (from ketoses via 2,3-enolization). The intermediate stage involves the degradation of these products through pH-dependent pathways: under acidic conditions (pH ≤ 7), 1,2-enolization produces 5-hydroxymethylfurfural or furfural, whereas under alkaline conditions (pH > 7), 2,3-enolization generates highly conjugated α-dicarbonyl compounds (O=C-C=O) that react with free amino acids via Strecker degradation. In the final stage, these α-dicarbonyl compounds conjugate with amino groups and other intermediates to form complex heterocyclic polymers, where aromatic ring hydrogens are substituted by hydroxyl, amino, and alkyl groups. Ultimately, these reactions yield heterogeneous nitrogen-containing polymeric pigments known as melanoidins [8,9]. The Maillard reaction scheme is shown in Scheme 1.

MR is an effective and environmentally friendly chemical modification technique. By forming conjugates through the covalent interaction of proteins and reducing sugars, MR can significantly improve emulsification, foaming, and solubility. For instance, Phoebe et al. [10] prepared whey protein isolate–beet pectin conjugates through MR, demonstrating approximately 20% higher solubility for the glycated products compared to native whey protein isolate. Additionally, the superior emulsifying capacity of WPI conjugates (EAI: 17.50–21.77 m^2^/g) over unmodified WPI (EAI: 5.22–18.48 m^2^/g) was demonstrated by Wang et al. [11]. The present state of research is predominantly oriented towards the investigation of bovine whey proteins, with comparatively limited attention devoted to the study of camel whey proteins (CWPs). Compared to bovine whey protein, CWP lacks β-lactoglobulin—the major allergen in cow’s milk. This unique composition confers hypoallergenic properties, making camel whey protein a promising alternative protein source for infant formulas and foods for special medical purposes, particularly for individuals with milk allergies. This study aims to establish a theoretical basis for creating innovative camel milk products in the food industry and to enhance the transformation of CWP into high-value-added products.

The study used CWP as the primary raw material to prepare camel whey protein–galactose conjugates (CWP-Gal) and camel whey protein–glucose conjugates (CWP-Glu). Investigations were conducted into how various reaction conditions affected the degree of MR between galactose and camel whey protein, as well as an in-depth analysis of the functional properties of CWP-Gal and CWP-Glu, including solubility, emulsification, foaming and surface hydrophobicity (H_0_). Furthermore, the structural changes in CWP were analyzed by studying sodium dodecyl sulfate–polyacrylamide gel electrophoresis (SDS-PAGE), intrinsic fluorescence spectrum, Fourier transform infrared spectroscopy (FT-IR), circular dichroism (CD), and scanning electron microscopy (SEM) to give CWP a theoretical foundation for bettering both its overall application and its quality.

## 2. Materials and Methods

### 2.1. Materials

Camel whey protein and walnut oil were provided by our laboratory. Galactose was produced from Shanghai Blue Season Technology Development Co., Ltd. (Shanghai, China). The ANS fluorescent probe (8-anilino-1-naphthalenesulfonic acid) and DTNB (5,5-dimercapto-2,2-dinitrobenzoic acid) were obtained from Shanghai Yuanye Biotechnology Co., Ltd. (Shanghai, China). Anhydrous ethanol and β-mercaptoethanol were produced by Fuchen Chemical Reagent Co., Ltd. (Tianjin, China). SDS-PAGE gel kit was purchased from Biosharp (Shanghai, China).

### 2.2. Preparation of Camel Milk Protein–Galactose and Camel Milk Protein–Glucose Conjugates

Camel whey protein was mixed with galactose (Gla) at mass ratios of 3:1, 2:1, 1:1, 1:2, and 1:3 (protein:Gla), vortexed thoroughly, and reacted at 95 °C for 3 h (pH = 9). The 1:1 protein–Gla mixture (pH = 9) was reacted under three conditions: 95 °C, 60 °C, and ultrasound-assisted (SB-3200DTD, Ningbo Scientz Biotechnology Co., Ltd., Ningbo, China) treatment at 60 °C (180 W, 30 min). Samples were taken hourly (1–5 h) and labeled. Untreated camel whey protein was labeled CWP and the camel whey protein–galactose conjugate was called CWP-Gal. In addition, CWP was mixed with glucose (Glu) in a mass ratio of 1:1 and reacted at 95 °C for 3 h (pH = 9) to obtain camel milk protein–glucose conjugate labeled as CWP-Glu. All the solutions after reaction were freeze-dried into dry powder for storage.

### 2.3. Degree of Grafting (DG)

The degree of glycosylation was quantitatively determined through spectrophotometric analysis using the o-phthalaldehyde (OPA) assay, which specifically detects free amino groups [12]. Prior to measurement, each protein sample (100 μL) was combined with 3 mL of OPA working solution and heated in carefully regulated water baths for precisely 2 min at 35 °C. The unmodified protein sample served as the negative control, and absorbance at 340 nm was measured using a 752N UV-visible spectrophotometer (Jinghua Technology Instrument Co., Shanghai, China). The degree of grafting was determined according to Equation (1):

(1) $$\mathrm{DG}(\%)=\frac{A_{c}-A_{s}}{A_{c}}\times 100$$

The calculation parameters were defined as follows: A_c_: absorbance of CWP; A_s_: absorbance of the glycoconjugated samples.

### 2.4. Degree of Browning

By measuring absorbance at 420 nm, the browning intensity of protein samples was determined, using a 752N UV-visible spectrophotometer (Shanghai Jinghua Technology Instrument Co., Ltd., Shanghai, China) [12].

### 2.5. Functional Properties

#### 2.5.1. Solubility

A MEGAFUGE 8R centrifuge (Thermo Fisher Scientific, Waltham, MA, USA) was used to centrifuge protein solutions (1 mg/mL) at 10,000× *g* for 15 min. Subsequent protein quantification was carried out using a NanoDrop spectrophotometer (Thermo Fisher Scientific, Wilmington, DE, USA) [13]. The solubility was computed according to Equation (2):

(2) $$\mathrm{Solubility}(\%)=\frac{C_{s}}{C_{0}}\times 100$$

The equation parameters were defined as follows: C_s_: protein content in the phase of supernatant; C_0_: initial protein concentration.

#### 2.5.2. Emulsifying Activity Index (EAI) and Emulsifying Stability Index (ESI)

Protein solutions (4 mL, 2 mg/mL) were emulsified with walnut oil (2 mL) using a homogenizer operating (Shanghai Lummy Mechanical Equipment Co., Ltd., Shanghai, China) at 14,000 rpm for 60 s. Five milliliters of a 0.1% (*w*/*v*) SDS solution were mixed with 50 microliters of emulsion aliquots that were taken at 0 and 10 min after homogenization, and vortexed thoroughly. Overall, 500 nm absorbance readings were obtained for calculating EAI (Equation (3)) and ESI (Equation (4)) [14].

(3) $$\mathrm{EAI}(m^{2}/g)=\frac{2\times 2.303\times A_{0}\times \mathrm{DF}}{\theta \times \varphi \times C\times 1000}$$

(4) $$\mathrm{ESI}(\min)=\frac{A_{0}\times 10}{A_{0}-A_{10}}$$

The equation parameters are defined as A_0_: initial emulsion absorbance (t = 0 min); A_10_: emulsion absorbance after 10 min incubation; DF: 100-fold dilution factor; *φ*: 1 cm cuvette light path length; θ: oil phase volume fraction (25%); C: protein concentration (2 mg/mL).

#### 2.5.3. Foaming Capacity (FC) and Foaming Stability (FS)

Using an LM-25 high-shear mixer (Shanghai Lummy Mechanical Equipment Co., Ltd., Shanghai, China), a 5 mL protein sample at 2 mg/mL concentration was homogenized in a 15 mL conical centrifuge tube at 18,000 rpm for 60 s. Immediately after foaming, the total volume was recorded to compute FC, followed by measuring FS after letting it stand for 30 min [15]. The determination of FC and FS was carried out by applying the mathematical relationships described in Equations (5) and (6):

(5) $$\mathrm{FC}(\%)=\frac{V_{0}-V_{i}}{V_{i}}\times 100$$

(6) $$\mathrm{FS}(\%)=\frac{V_{30}-V_{i}}{V_{0}-V_{i}}\times 100$$

The equation parameters are defined as V_i_: Initial measurement; V_0_: Post-homogenization; V_30_: After 30 min foam settling.

### 2.6. Structural Properties

#### 2.6.1. Sodium Dodecyl Sulfate–Polyacrylamide Gel Electrophoresis (SDS-PAGE)

Polyacrylamide gels were prepared using Biosharp Gel Rapid Preparation Kit following manufacturer protocols. Protein samples (2 mg/mL) were heat-denatured in loading buffer containing 2% β-mercaptoethanol (reducing conditions) at 100 °C for 8 min. Then, 20 μL aliquots were electrophoresed on 12% SDS-PAGE gels using Tris-glycine running buffer at constant voltages of 90 V for the stacking gel and 120 V for the separating gel [16].

#### 2.6.2. Surface Hydrophobicity (H_0_)

A dilution gradient of protein solutions (0.01–0.4 mg/mL) was established, with each 4 mL aliquot incubated with 50 μL of 8 mM ANS fluorescent probe. The mixtures were protected from light and incubated for 15 min to allow for ANS binding. Fluorescence emission spectra (400–600 nm) were recorded with 390 nm excitation using an Agilent spectrophotometer (Agilent Technologies, Santa Clara, CA, USA). The maximum fluorescence intensity at each protein concentration was recorded, and the protein concentration-fluorescence intensity regression slope was used to calculate surface hydrophobicity [17].

#### 2.6.3. Intrinsic Fluorescence Spectrum

Using the fluorescence spectrophotometer (Agilent Technologies, Santa Clara, CA, USA), intrinsic fluorescence spectra were obtained. The wavelengths for excitation and emission were set at 292 nm and 300–450 nm, respectively, to determine the experimental conditions [18].

#### 2.6.4. Fourier Transform Infrared (FT-IR)

FT-IR spectroscopic analysis was performed on the samples using an FT-IR spectrophotometer (PerkinElmer, Waltham, MA, USA). The FT-IR spectra were collected in the range of 4000–400 cm^−1^ with a resolution of 4 cm^−1^, accumulating 32 scans per measurement [17].

#### 2.6.5. Circular Dichroism (CD)

Circular dichroism (CD) spectra (190–250 nm) of the protein solutions (0.5 mg/mL) were obtained using a spectropolarimeter (Jasco International Co., Ltd., Tokyo, Japan). The spectra were averaged over 3 scans with a 1 nm bandwidth and a scan speed of 50 nm/min [11]. Secondary structure analysis was performed using CDNN software (version 2, Applied Photophysics Ltd., Leatherhead, UK).

#### 2.6.6. Scanning Electron Microscope (SEM)

Sample morphology was examined by SEM (SU-8010, Hitachi High-Technologies, Tokyo, Japan) operating at 5 kV accelerating voltage [19].

#### 2.6.7. Statistical Analysis

SPSS 27.0 (SPSS Inc., Chicago, IL, USA) was used for ANOVA to detect significant mean differences (*p* < 0.05).

## 3. Results and Discussion

### 3.1. Analysis of Degree of Grafting (DG) and Browning Intensity

**Figure 1:**
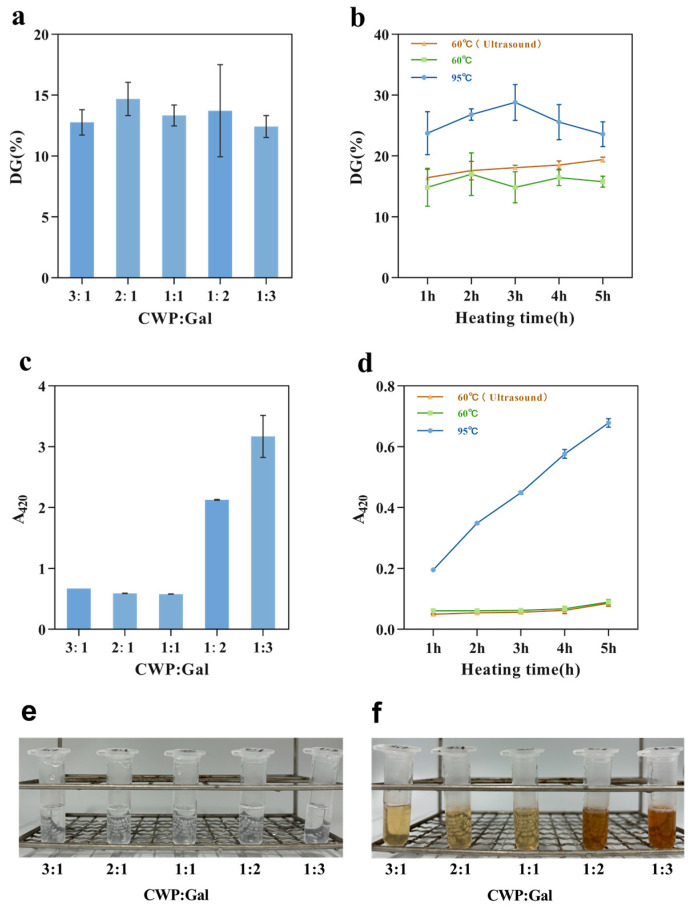
Impact of varying reaction conditions on MR. (**a**) DG with different mass ratios of CWP:Gal; (**b**) DG with ultrasound, different reaction times, and different temperature treatments; (**c**) browning degree across CWP and Gal mass ratios; (**d**) browning degree under ultrasound, reaction time, and temperature variations; (**e**) pre-MR color at various CWP and Gal mass ratios; (**f**) post-MRcolor at various CWP and Gal mass ratios.

**Figure 2:**
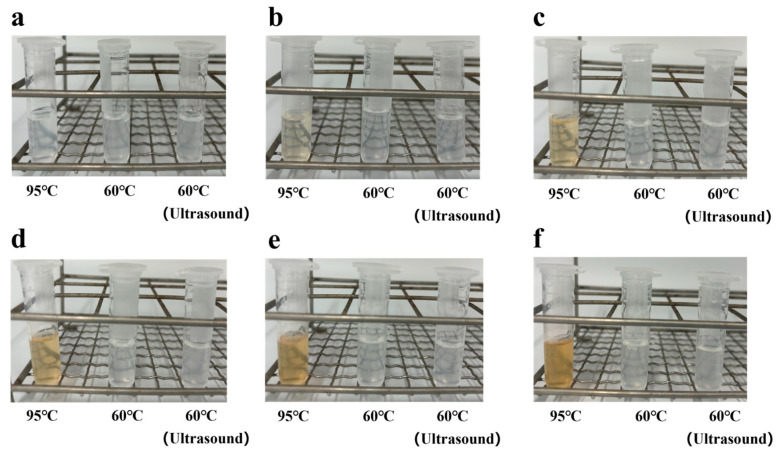
Color changes during MR between CWP and Gla under three conditions of 5 h: 95 °C, 60 °C, and 60 °C with ultrasound. Time points: (**a**) 0 h; (**b**) 1 h; (**c**) 2 h; (**d**) 3 h; (**e**) 4 h; (**f**) 5 h.

The progression of Maillard reaction at various galactose-to-CWP mass ratios appears in Figure 1a,c, where absorbance measurements at 420 nm reveal MR end product formation [20]. The results showed that the 1:1 ratio exhibited the lowest browning intensity (Figure 1c), with visual color changes shown in Figure 1e,f. The degree of grafting reached its maximum at the 2:1 ratio, while the 1:1 ratio showed no statistically significant difference in grafting degree compared to the 2:1 ratio. Figure 1b shows that the grafting degree between CWP and Gla peaked at 28% after 3 h of MR at 95 °C. A similar trend was observed at 60 °C, the grafting degree peaked at 17% after 2 h of MR. With prolonged Maillard reaction time, the spatial structure of protein molecules unfolded, exposing more free amino groups and promoting covalent conjugation with sugar molecules. This initially enhanced the reaction extent, followed by a subsequent decrease likely caused by prolonged heating, which induced protein aggregation and intermolecular cross-linking [21]. CWP-Gal exhibits a substantially higher grafting degree (28%) compared to the whey protein–gum arabic conjugate (8%) [22]. This difference in covalent modification efficiency directly correlates with CWP-Gal’s improved functional performance, including enhanced solubility and emulsification stability. Under ultrasound treatment at 60 °C, DG increased progressively with reaction time, reaching 19% after 5 h of MR. This enhancement is attributed to ultrasound-induced protein unfolding, which increased the accessibility of free amino groups for conjugation [23]. As shown in Figure 1d, the browning intensity progressively increases with reaction time at 95 °C, which correlates with the visual color changes depicted in Figure 2. This provides a comprehensive characterization of the Maillard reaction, beginning with the initial stage where unstable Schiff bases form (no visible browning observed), progressing to the intermediate stage marked by the first visible browning and formation of early melanoidin precursors, and culminating in the final stage with production of nitrogen-containing brown polymeric melanoidins [8,24].

In food systems, browning is often accompanied by changes in flavor and nutrients, and low browning products better preserve the original flavor and nutrition [25]. Therefore, subsequent experiments were performed using the product obtained at 95 °C for 3 h with a 1:1 ratio, selected based on high grafting degree and low browning criteria. For comparison, CWP-Glu (the DG of 7%) prepared under identical MR conditions were analyzed in parallel to evaluate the differential effects of Gal and Glu in the MR modification of CWP.

### 3.2. Effect of MR on Functional Properties of CWP

#### 3.2.1. Solubility

**Figure 3:**
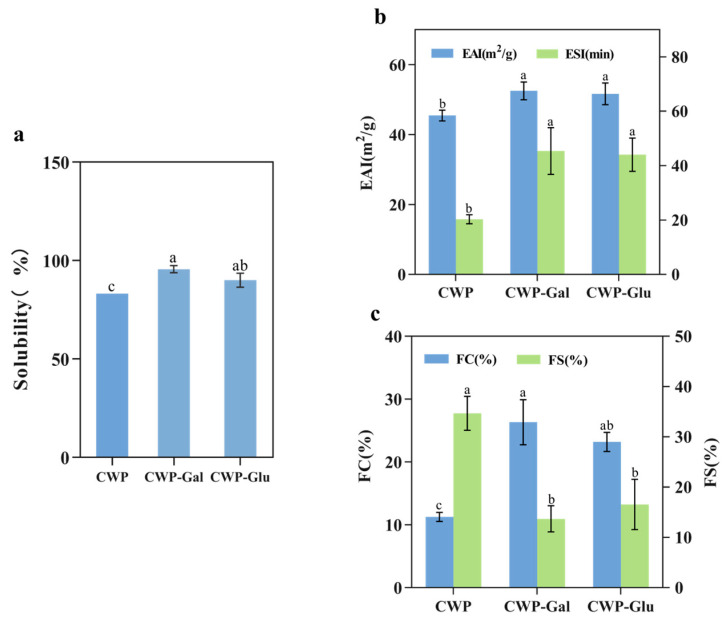
Functional properties of CWP, CWP-Gal, and CWP-Glu. (**a**) Solubility; (**b**) EAI and ESI; (**c**) FC and FS. Groups with distinct superscript letters exhibit significant differences (*p* < 0.05).

Figure 3a demonstrates that both CWP-Gal and CWP-Glu exhibited significantly enhanced solubility compared to native CWP, with increases of 14.90% and 8.17%, respectively (*p* < 0.05). The enhanced solubility results from covalent conjugation of hydrophilic sugar moieties during MR, which increases surface polarity of the protein conjugates through exposed hydroxyl groups [21]. CWP-Gal exhibited 5.60% higher solubility than CWP-Glu. The elevated glycosylation degree in CWP-Gal conjugates reflects enhanced covalent attachment of galactose molecules to camel whey protein through MR, increasing hydrophilic groups on its surface. This abundance of hydrophilic hydroxyls reduces protein polymerization due to spatial barriers and electrostatic repulsion [19]. Aligning with emulsification test results, MR enhances protein solubilization, raising protein concentration and mobility in aqueous solutions, thereby improving adsorption at the oil/water interface and enhancing emulsifiability [26]. Compared to the solubility of bovine whey protein–dextran conjugate (86%), CWP-Gal and CWP-Glu exhibited enhanced solubility with increases of 10.47% and 4.65%, respectively, demonstrating their superior dispersibility in aqueous systems [27].

#### 3.2.2. Emulsifying and Foaming Properties

As shown in Figure 3b, Maillard conjugation with either galactose or glucose (CWP-Gal/Glu) greatly enhanced both EAI and ESI relative to unmodified CWP (*p* < 0.05). Glycation of CWP with Gal via Maillard reaction increased EAI by 15.53% and extended ESI from 20 to 45 min. Through their amphipathic nature, protein molecules adsorb at oil/water interfaces and subsequently organize into interfacial thin-film architectures. Due to their exceptional solubility in aqueous conditions, sugar molecules also perform a crucial protective role in the aforementioned systems [26]. The emulsification activity of CWP-Gal was increased by 13.64% compared to CWP, and the emulsification stability was improved from 20 min to 44 min. The ESI of bovine whey protein–xylose conjugates measured 14.5 min, compared to 45 min (CWP-Gal) and 44 min (CWP-Glu), demonstrating camel whey protein conjugates’ superior emulsion stabilization capacity [28]. The above studies suggest that Gla and Glu play a favorable role in CWP modification.

We evaluated FC and FS of CWP and its Maillard conjugates (CWP-Gal/Glu). CWP-Gal showed 113.95% higher FC versus native CWP, while CWP-Glu exhibited 106.03% increase (Figure 3c, *p* < 0.05). The hydrophilic groups introduced on the molecules during protein MR increase the molecular flexibility and electrostatic repulsion between the molecules, thus reducing the size of the aggregates [29]. Concurrently, MR-induced unfolding of α-helix structures exposed additional hydrophobic groups, the adsorption of modified proteins at the air-water interface was enhanced during foaming, leading to improved foaming capacity [30]. In addition, both conjugates showed reduced FS compared to native CWP. FS decreased from 34.70% to 13.70% for CWP-Glu and from 34.7% to 16.55% for CWP-Gal (*p* < 0.05). The observed destabilization arises from the synergistic effects of glycosylation-induced steric constraints and increased polypeptide backbone rigidity, which collectively disrupt the self-assembly and self-repair mechanisms of interfacial protein films [31].

### 3.3. Effect of MR on Structural Properties of CWP

#### 3.3.1. SDS-PAGE

**Figure 4:**
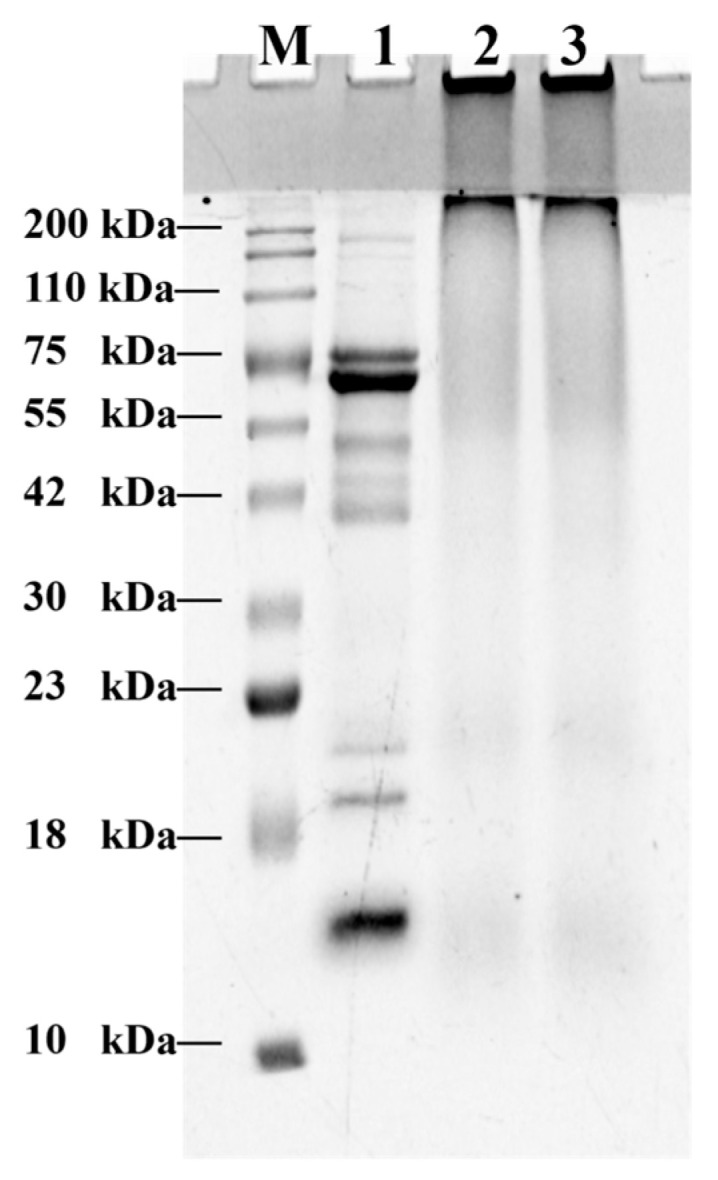
SDS-PAGE plots of CWP, CWP-Gal and CWP-Glu. M: Maker; 1: CWP; 2: CWP-Gal; 3: CWP-Glu.

Electrophoretic separation revealed three predominant protein components in CWP (Figure 4): lactoferrin (75 kDa), serum albumin (66 kDa), and α-lactalbumin (13 kDa). The successful conjugation of CWP with galactose and glucose was confirmed by SDS-PAGE, as evidenced by characteristic band shifts. As depicted in Figure 4, a new band can be observed at the top of lanes 3 and 4, which clearly shows the formation of CWP-Gal and CWP-Glu, but the molecular weight is too large to migrate into the gel. Electrophoretic analysis revealed marked attenuation of original protein bands in both CWP-Glu and CWP-Gal conjugates relative to native CWP, accompanied by noticeable band broadening across the 13–80 kDa molecular weight range. The diffuse nature of the MR product bands suggests covalent binding of protein molecules to varying amounts of reducing sugars [32].

#### 3.3.2. Surface Hydrophobicity (H_0_) Analysis

**Figure 5:**
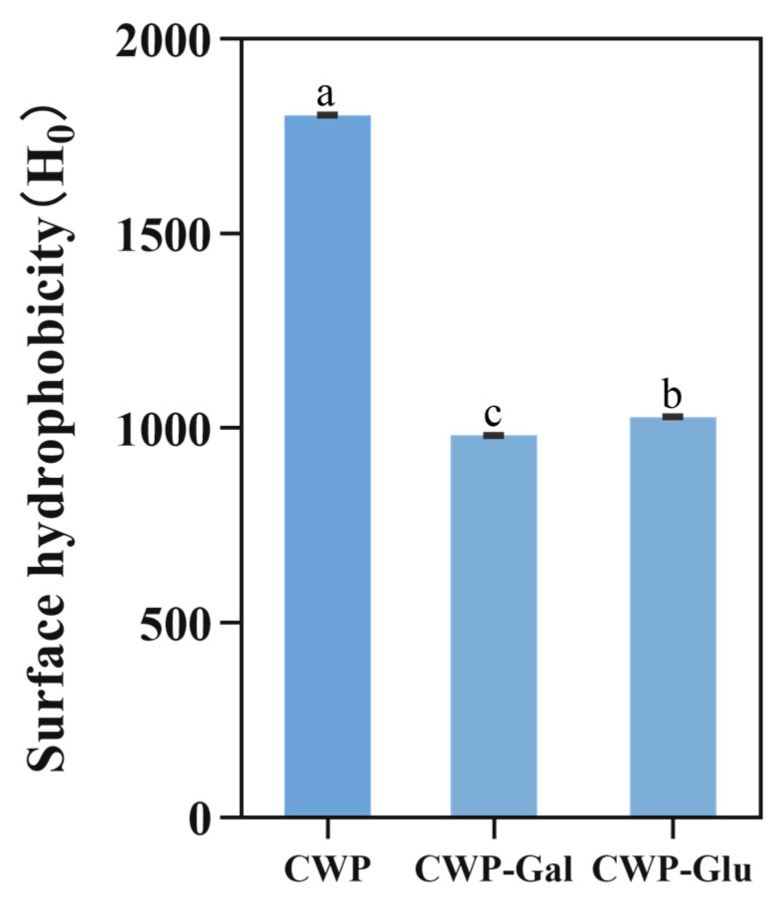
Surface hydrophobicity of CWP, CWP-Gal and CWP-Glu. Groups with distinct superscript letters exhibit significant differences (*p* < 0.05).

The surface hydrophobicity of CWP, CWP-Gal and CWP-Glu is shown in Figure 5. The H_0_ of untreated CWP was 1804, and the H_0_ was significantly reduced to 981 and 1029 after grafting of CWP with Gal and Glu, respectively. Comparatively, bovine whey protein–hyaluronic acid conjugate showed an H_0_ value of 1250. These results demonstrate that both CWP-Gal and CWP-Glu possess enhanced hydrophilic character relative to both the native CWP and the bovine protein conjugate [33]. The spatial blocking effect of sugar molecules adhering to proteins after Maillard reaction reduces surface hydrophobic group exposure, preventing ANS proximity to hydrophobic regions and decreasing H_0_ [22]. The attachment of hydrophilic sugar molecules increases surface hydrophilicity of the molecule, thereby minimizing exposure of internal hydrophobic groups [34]. Although it has been suggested that a higher degree of glycation may lead to a more significant decrease in H_0_ [35], the present study observed that CWP-Glu with a higher glycation degree exhibited significantly higher H_0_ values than CWP-Gal (*p* < 0.05). This apparent discrepancy may originate from the distinct structural properties of the two reducing sugars, as galactose moieties demonstrate stronger steric hindrance effects per conjugated residue despite lower conjugation stoichiometry.

#### 3.3.3. Intrinsic Fluorescence Spectrum

**Figure 6:**
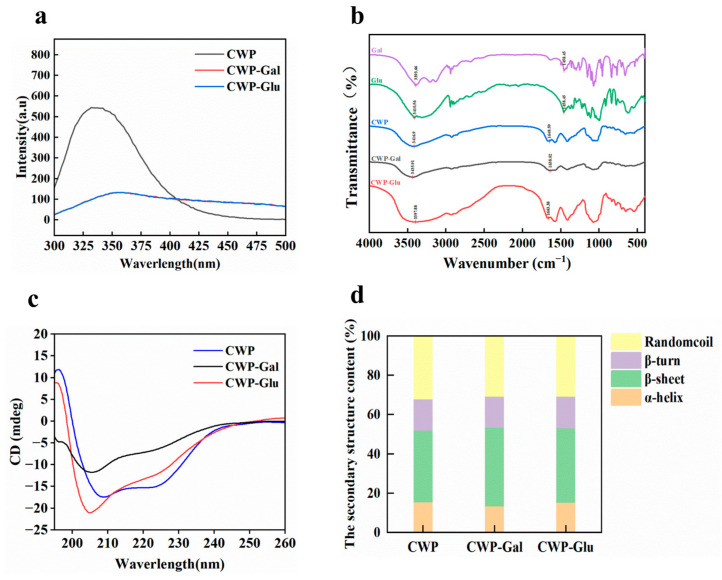
Structural characterization of CWP, CWP-Gal and CWP-Glu. (**a**) Intrinsic fluorescence spectra; (**b**) FT-IR spectra; (**c**) circular dichroism; (**d**) secondary structure content.

As shown in Figure 6a, CWP-Gal and CWP-Glu showed significantly lower fluorescence intensit compared to CWP. The emission peak of CWP is located at 344 nm, while the emission peaks of CWP-Gal and CWP-Glu are located at 360 nm, indicating a “red-shift” phenomenon. During Maillard reaction, sugar compounds alter protein molecular conformation, specifically forming flexible irregularly curled structures. These structural changes increase spatial site-blocking effects between molecules, effectively masking tryptophan residue fluorescence and decreasing overall fluorescence intensity. This decrease in fluorescence intensity is further influenced by the covalent grafting of Gal and Glu onto CWP, which induces a “masking” effect [36].

#### 3.3.4. Fourier Transform Infrared (FT-IR)

The protein exhibited characteristic amide I (1700–1600 cm^−1^) and amide II (1550–1500 cm^−1^) absorption bands in FT-IR spectra [37]. While the amide II mode combines C-N stretching with N-H bending vibrations, the amide I region is mostly the result of C=O bond stretching. Additionally, the signals observed between 3600 cm^−1^ and 3200 cm^−1^ represent N-H and O-H stretching vibrations, indicative of intermolecular and intramolecular hydrogen bonding [38].

The observed red shift in the amide I band absorption peak from 1648.59 cm^−1^ to 1638.02 cm^−1^ in CWP-Gal (Figure 6b) clearly demonstrates the formation of covalent bonds between the amino groups of CWP and the carbonyl groups of Glu, accompanied by a reduction in -NH groups. While the absorption band near 1650 cm^−1^ is characteristic of Schiff base-derived C=N bonds, the shifted band at 1638 cm^−1^ likely represents a superposition of C=O and C=N vibrational modes, confirming the formation of Maillard reaction products (MRPs) through unstable Schiff base intermediates between CWP and Gal. The structural modification tailored the protein’s surface charge distribution, thereby improving interfacial activity, with a corresponding increase in emulsification activity index (EAI) [39,40]. The O-H/N-H stretching vibration of CWP at 3416.90 cm^−1^ exhibited a 19.01 cm^−1^ blueshift to 3435.91 cm^−1^ following galactosylation, indicative of newly introduced hydroxyl groups from covalently conjugated galactose residues [30]. The broadened vibrational envelope (3600–3200 cm^−1^) observed in CWP-Glu conjugates, compared to native CWP, originates from intensified N-H/O-H stretching modes resulting from glucose-derived hydroxyl groups incorporated via covalent grafting. The observed modifications in amide I/II band intensities provide evidence for the structural rearrangement into stable Amadori products, characterized by the formation of additional C=O and C-N bonds. This transformation enhances the molecular architecture through increased steric stabilization at oil/water interfaces, ultimately leading to improved emulsion stability [41].

#### 3.3.5. Circular Dichroism

Circular dichroism analysis (Figure 6c,d) demonstrates MR-induced modifications to CWP secondary structure. The CD spectral signal absorption of CWP-Gal and CWP-Glu was further changed following MR, as shown in Figure 6c. There were notable shifts in the peak shapes and changes in the position of the peaks, suggesting that the sugar molecules covalently bonded with the CWPs, changing the number and position of hydrogen bonds within the original molecules and, as a result, affecting the secondary structure [42]. Figure 6d shows that α-helix (15.47%), β-sheet (36.58%), β-turn (16.03%), and Random coil (31.90%) are all present in the secondary structure of CWP. Condensation reactions between galactose and ε-amino groups within or adjacent to α-helix domains likely contributed to the observed 12.47% reduction in α-helix content in galactosylated CWP. CWP-Gal exhibited a 9.70% increase in β-sheet content, while random coil and β-turn structures showed reductions to 30.63% and 15.66%, respectively. the proportion of β-turn of CWP-Glu was elevated by 3.58%, which had a lesser effect on the other secondary structures. the Gal and Glu conjugates with CWP showed different structural changes, and these phenomena indicated that the sugar molecule binding changed the original conformation of CWP [43].

#### 3.3.6. Scanning Electron Microscope (SEM)

**Figure 7:**
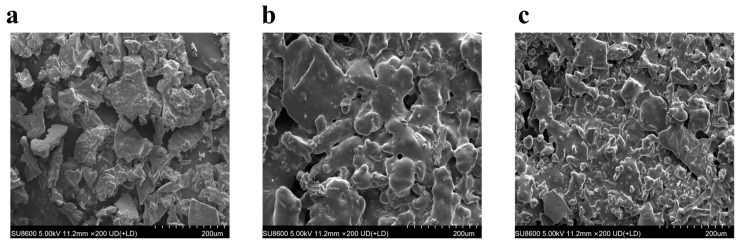
SEM images of CWP, CWP-Glu, and CWP-Gal. (**a**) CWP; (**b**) CWP-Glu; (**c**) CWP-Gal. Magnification is 200×, scale bar = 200 μm.

Figure 7 illustrates the scanning electron microscope images of CWP, CWP-Glu, and CWP-Gal. Observations show that the untreated CWP exhibits a blocky microstructure with a smooth surface [44]. In contrast to CWP, the structures of CWP-Gal and CWP-Glu showed differing levels of relaxation, and fracture was noted, resulting in fragments of different sizes. Microstructural analyses reveal that MR induces significant CWP conformational changes, manifested as gradual spatial expansion, and fragmentation. These morphological transformations, likely mediated by galactose and glucose incorporation, generate novel architectural features.

## 4. Conclusions

CWP-Gal and CWP-Glu were successfully prepared in this study, and by analyzing the effects of temperature, sonication, reaction time, and the protein to galactose mass ratio on the extent of MR, the ideal reaction conditions for protein and galactose were found to be 95 °C, a 1:1 mass ratio, and 3 h of reaction. In terms of functional properties, relative to CWP, CWP-Gal increased the solubility by 14.90%, while CWP-Glu increased by 8.17%; the emulsifying activity of CWP-Gal increased by 15.53%, and that of CWP-Glu increased by 13.64%; the emulsification stability of CWP-Gal was increased from 20 to 45 min, and that of CWP-Glu to 44 min; the foaming ability of CWP-Gal was enhanced by 113.95% and that of CWP-Glu by 106.03%; the foam stability of CWP-Gal was lowered from 34.70% to 13.70% and that of CWP-Glu from 34.7% to 16.55%. In conclusion, it is evident that CWP-Gal outperforms CWP-Glu in terms of solubility, foaming, and emulsifying qualities. Structural analysis showed that the α-helix of CWP-Gal decreased by 12.47% and the β-sheet increased by 9.70%, whereas the β-sheet of CWP-Glu increased by 3.58%. Regarding surface hydrophobicity (H_0_), CWP-Gal decreased from 1804 to 981, and CWP-Glu decreased to 1029. The results of intrinsic fluorescence spectroscopy, FT-IR, and SEM further confirmed the successful covalent binding between sugar molecules and proteins, as well as microstructural changes in CWP. These results indicate that Gal modification can significantly improve the functional properties and structural stability of CWP, providing a foundation for producing high-value dairy protein ingredients. The improved solubility and emulsification of CWP-Gal suggest its potential as a functional ingredient in acidic beverages (e.g., camel milk-based probiotic drinks), infant formulas, where bovine whey alternatives are sought. Future studies should evaluate its stability in these matrices.

## Data Availability

The original contributions presented in the study are included in the article; further inquiries can be directed to the corresponding author.
